# Resting-state functional connectivity of hippocampal subregions in predicting escitalopram treatment outcome in major depressive disorder

**DOI:** 10.3389/fpsyt.2026.1946797

**Published:** 2026-09-30

**Authors:** Fengxia Zhang, Xiaoya Li, Ling Zhang, Yuan Zhou, Jingjing Zhou, Yuan Feng, Gang Wang, Aihong Yu, Yun Wang

**Affiliations:** 1 Department of Radiology, Beijing Anding Hospital, Capital Medical University, Beijing, China; 2 Beijing Key Laboratory of Intelligent Drug Research and Development for Mental Disorders, National Clinical Research Center for Mental Disorders, National Center for Mental Disorders; Beijing Anding Hospital, Capital Medical University, Beijing, China; 3 Advanced Innovation Center for Human Brain Protection, Capital Medical University, Beijing, China; 4 State Key Laboratory of Cognitive Science and Mental Health, Institute of Psychology, Beijing, China; 5 Department of Psychology, University of Chinese Academy of Sciences, Beijing, China

**Keywords:** escitalopram, functional connectivity, hippocampal subregions, major depressive disorder, resting-state fMRI

## Abstract

**Objective:**

To examine whether pretreatment resting-state functional connectivity (rsFC) of hippocampal subregions is associated with 12-week escitalopram treatment outcomes in major depressive disorder (MDD).

**Methods:**

A total of 87 patients with MDD received 12 weeks of escitalopram monotherapy and underwent resting-state functional MRI before treatment. Based on the 17-item Hamilton Depression Rating Scale (HAMD-17) score at week 12, patients were classified into the remission depression group (RD, n = 42) or the nonremission depression group (NRD, n = 45). Seed-based whole-brain rsFC analyses were performed using eight bilateral hippocampal subregions as seeds. Group differences in rsFC were examined, and exploratory logistic regression and receiver operating characteristic analyses were conducted to assess the discrimination of remission. Partial correlations between differential rsFC and the HAMD-17 reduction rate were calculated controlling for age, sex, and education level.

**Results:**

Compared with NRD, RD showed higher pretreatment rsFC between the left hippocampal tail and the right inferior parietal lobule and lower rsFC between the right hippocampal body and the left middle occipital gyrus and between the right hippocampal tail and the right cuneus. An exploratory combined model integrating the three differential rsFC measures showed discrimination between the RD and NRD groups, with an area under the curve of 0.841 (95% confidence interval: 0.755–0.915). The HAMD-17 reduction rate was negatively associated with right hippocampal body–left middle occipital gyrus rsFC and right hippocampal tail–right cuneus rsFC, but positively associated with left hippocampal tail–right inferior parietal lobule rsFC.

**Conclusion:**

Patterns of pretreatment rsFC involving the hippocampal body and tail and parietal and visual cortical regions were associated with 12-week escitalopram treatment outcomes in MDD. These connectivity measures may represent candidate imaging features for further investigation, although validation in larger independent samples is required.

## 1 Introduction

Major depressive disorder (MDD) is a common psychiatric disorder characterized primarily by persistent depressed mood and anhedonia (1). Affecting approximately 185 million people worldwide, MDD is associated with impaired functioning and reduced quality of life and contributes substantially to the global burden of disease (2). Although antidepressant medications constitute a mainstay of treatment for MDD, clinical outcomes vary considerably across individuals, and a substantial proportion of patients do not achieve remission with initial pharmacological treatment (3). The neurobiological basis underlying this variability remains incompletely understood. Accordingly, investigating the relationship between pretreatment brain functional characteristics and subsequent treatment outcomes may help clarify the neural mechanisms associated with antidepressant response and provide neuroimaging evidence relevant to treatment efficacy (4, 5).

MDD has been associated with altered functional interactions among distributed neural systems involved in affective and cognitive processing (6). The functional organization of these systems may also be relevant to individual differences in antidepressant treatment outcomes. Previous resting-state functional connectivity (rsFC) studies have linked pretreatment functional connectivity to subsequent antidepressant outcomes. For example, baseline connectivity within and between large-scale brain networks differed between remitters and non-remitters following antidepressant treatment (4). Pretreatment connectivity patterns involving the frontoparietal and default mode networks were also associated with response to escitalopram (5). More recently, baseline functional connectivity between the subgenual anterior cingulate cortex and frontoparietal regions was associated with remission and symptom improvement after escitalopram treatment (7). Together, these findings suggest that pretreatment functional connectivity may capture neural circuit features associated with subsequent treatment outcomes.

The hippocampus is critically involved in episodic and emotional memory and stress-related processes and is extensively connected with cortical and limbic regions supporting affective and cognitive functions, making it highly relevant to the pathophysiology of MDD (8, 9). A large-scale multicenter study by Schmaal et al. (10) identified reduced hippocampal volume as one of the most robust subcortical structural abnormalities in MDD. Furthermore, Travis et al. (11) reported that cortisol levels were associated with hippocampal subfield volumes and memory performance, suggesting a relationship between stress-related biological processes and hippocampal structural alterations. Resting-state functional magnetic resonance imaging (fMRI) studies have further demonstrated that hippocampal connectivity abnormalities in MDD are regionally heterogeneous. Cao et al. (12) found disrupted hippocampal functional connectivity with frontal, parietal, and cerebellar regions, some of which was related to illness duration. Hao et al. (13) showed that the cornu ammonis (CA), dentate gyrus, and subiculum exhibited distinct whole-brain functional connectivity abnormalities involving the premotor cortex, insula, orbitofrontal cortex, and ventrolateral prefrontal cortex. Wu et al. (14) also demonstrated that hippocampal connectivity abnormalities are subregion-specific and sensitive to imaging modality and temporal scale, with prominent alterations in the CA region, particularly the right CA1. Collectively, structural and functional neuroimaging evidence supports widespread and regionally heterogeneous hippocampal abnormalities in MDD, implicating the hippocampus and its associated circuits in disease pathophysiology.

Beyond their involvement in the pathophysiology of MDD, structural and functional characteristics of the hippocampus have also been closely associated with antidepressant treatment outcomes. Structural neuroimaging studies suggest that both pretreatment hippocampal volume and treatment-related volumetric changes in hippocampal subregions may be associated with subsequent clinical outcomes. For example, Tai et al. (15) found that antidepressant medication and cognitive behavioral therapy produced distinct longitudinal patterns of volumetric change in the hippocampal tail and other subfields, and that the baseline volumes of certain hippocampal subfields were associated with clinical improvement. Kamishikiryo et al. (16) further reported that escitalopram responders had a larger left hippocampal volume before treatment and showed greater increases in the volumes of the right hippocampus and right hippocampal head following treatment. A multimodal neuroimaging study similarly showed that larger bilateral hippocampal volumes were associated with better pharmacological outcomes, while functional connectivity patterns involving the right temporal and parahippocampal regions measured shortly after hospital admission were associated with subsequent symptom improvement (17). At the whole-hippocampus and large-scale network levels, Xiao et al. (18) found that patients showing early improvement after escitalopram treatment exhibited greater pretreatment connectivity of the left hippocampus with the left inferior frontal gyrus and precuneus, with both connections being positively associated with symptom reduction. Gong et al. (19) reported that lower nodal efficiency of the left hippocampus was associated with greater short-term symptom improvement. The EMBARC study further demonstrated that pretreatment connectivity between the hippocampus and other functional networks moderated sertraline and placebo outcomes in opposite directions (20). In late-life depression, greater right orbitofrontal cortex–left hippocampus connectivity was associated with lower post-treatment depression severity, whereas greater posterior cingulate cortex–left hippocampus connectivity was associated with greater symptom reduction over time (21). In addition, Zhang et al. (22) identified pretreatment differences between responders and nonresponders after 2 weeks of selective serotonin reuptake inhibitor (SSRI) treatment in regional homogeneity of the right parahippocampal gyrus and functional connectivity involving temporal regions. Collectively, these studies support an association between hippocampal and parahippocampal circuits and antidepressant treatment outcomes.

Further evidence suggests that distinct hippocampal subregions may play different roles in antidepressant treatment. Regarding longitudinal treatment-related changes, Bai et al. (23) found increased functional connectivity of the hippocampal emotion-related subregion with the left middle occipital gyrus and right middle temporal gyrus following electroconvulsive therapy (ECT), with greater increases in hippocampal emotion-related subregion–right middle temporal gyrus connectivity being associated with greater improvement in depressive symptoms. Following esketamine treatment, increased functional connectivity was also observed between the right caudal hippocampus and the left cerebellar lobule VI, precuneus, and middle temporal gyrus (24). These longitudinal studies demonstrate that hippocampal subregional connectivity can change in response to treatment; however, whether pretreatment connectivity of specific hippocampal subregions is associated with subsequent treatment outcomes remains less well established. Zhang et al. (25) reported that patients who achieved remission following repeated ketamine infusions showed less negative pretreatment functional connectivity of the left rostral hippocampus with the right angular gyrus, left inferior parietal cortex, and right superior parietal cortex than nonremitters; these baseline connectivity measures also showed discriminative value for subsequent remission. Xue et al. (26) found that subsequent responders exhibited lower baseline dynamic functional connectivity of the left rostral hippocampus, involving connections with the left superior temporal gyrus and right precentral gyrus, and that this measure was negatively correlated with clinical improvement. Kuai et al. (27) further reported that baseline dynamic functional connectivity between the left rostral hippocampus and right precentral gyrus was negatively associated with clinical improvement after 3 months and mediated the association between left rostral hippocampal volume and antidepressant efficacy. Overall, previous findings support an association between hippocampal subregional functional connectivity and antidepressant outcomes, but substantial heterogeneity remains in hippocampal parcellation schemes, connected regions, and directions of association. Evidence remains limited regarding whether pretreatment whole-brain functional connectivity of hippocampal subregions is associated with subsequent remission and symptom improvement under a uniform pharmacological treatment protocol.

Against this background, we conducted seed-based whole-brain rsFC analyses using bilateral hippocampal subregions as separate seeds to determine whether connectivity involving any of these subregions was associated with 12-week escitalopram treatment outcomes in patients with MDD. Escitalopram was selected because it is recommended as a first-line pharmacological treatment for MDD and has demonstrated a favorable balance of efficacy and acceptability (28). In addition, escitalopram is a highly selective serotonin reuptake inhibitor with a well-characterized pharmacological profile (29). Specifically, hippocampal subregions were defined using the Scale III (S3) parcellation of the Melbourne Subcortex Atlas, which was developed by Tian et al. (30) based on spatial gradients in rsFC and divides each hippocampus into the medial head, lateral head, body, and tail. Given that clinical improvement may emerge later in the treatment course in some patients, the 12-week follow-up allowed later-onset improvement to be captured and provided a more comprehensive assessment of acute treatment outcomes (31). At week 12, patients were classified into remission depression (RD) and nonremission depression (NRD). We compared baseline rsFC between the RD and NRD groups, examined associations between differential rsFC measures and reductions in depressive symptoms, and evaluated the discriminative ability of these rsFC measures for remission status.

## 2 Methods

### 2.1 Participants

A total of 110 MDD outpatients were recruited at Beijing Anding Hospital, Capital Medical University, between July 2018 and June 2021. All participants were assessed by psychiatrists, and the diagnosis of MDD was established using the Mini International Neuropsychiatric Interview (M.I.N.I.) 5.0.0 based on the Diagnostic and Statistical Manual of Mental Disorders, Fourth Edition (DSM-IV) criteria. Patients were eligible for inclusion if they met the following criteria: (1) met DSM-IV criteria for MDD, with either a first or recurrent depressive episode and without psychotic features; (2) a score of ≥ 14 on the 17-item Hamilton Depression Rating Scale (HAMD-17); (3) drug-naïve or had taken no more than 7 days of antidepressant medication in the 14 days prior to the study; and (4) aged 18–65 years, Han Chinese ethnicity, right-handedness, and completion of at least primary school education. Patients were excluded if they met any of the following criteria: (1) a current or lifetime history of any psychiatric disorder other than MDD; (2) a history of alcohol or substance abuse or dependence; (3) a major physical illness or organic brain disease; (4) severe suicidal ideation or a history of suicide attempts; (5) pregnancy or breastfeeding; (6) receipt of electroconvulsive therapy within 3 months before enrollment; (7) a history of intolerance or nonresponse to escitalopram; or (8) any contraindication to magnetic resonance imaging.

Patients were withdrawn from the study if they violated the medication protocol during treatment, including the use of other antidepressants or mood stabilizers or receipt of combination antidepressant therapy, or if they experienced serious adverse drug reactions. The study protocol was approved by the Medical Ethics Committee of Beijing Anding Hospital, Capital Medical University (approval no. 2017-24). The study cohort was registered with the Chinese Clinical Trial Registry (registration number: ChiCTR-OOC-17012566). All patients participated voluntarily and provided written informed consent before enrollment. The present study used the same cohort and final analytic sample as our previous publication (7). In this study, we addressed different hypotheses, and the analyses reported here do not overlap with those previously published.

### 2.2 Treatment and clinical outcome assessment

All patients received escitalopram treatment for 12 weeks. Escitalopram was initiated at 5 mg/day and titrated to a therapeutic dose of 10–20 mg/day within the first week. Thereafter, the dosage was individually adjusted within this range by the treating psychiatrist according to clinical response and tolerability. Throughout the treatment period, experienced psychiatrists reviewed each patient’s prescribed regimen and self-reported medication use during follow-up. Medications for insomnia were permitted when clinically necessary, whereas other psychotropic medications were not allowed throughout the treatment period. Thirteen patients did not complete the 12-week follow-up because of loss to follow-up, protocol deviations, or changes in medication, leaving 97 patients who completed the treatment protocol and had available pretreatment MRI data. Depressive symptom severity was assessed using the HAMD-17 at baseline and at week 12. Remission was defined as a HAMD-17 score ≤7 at the end of treatment, and patients were accordingly categorized into the RD and NRD groups. The percentage reduction in HAMD-17 scores was calculated as [(baseline HAMD-17 score − week-12 HAMD-17 score)/baseline HAMD-17 score] × 100%.

### 2.3 MRI data acquisition

Magnetic resonance imaging (MRI) data were collected at baseline using a 3.0 T Siemens Prisma whole-body scanner equipped with a 64-channel head coil at Beijing Anding Hospital, Capital Medical University. During resting-state scanning, participants were instructed to remain still with their eyes closed, stay awake, and not to think of anything in particular. Resting-state functional images were acquired using an echo-planar imaging sequence with the following parameters: repetition time (TR) = 2000 ms, echo time (TE) = 30 ms, field of view (FOV) = 200 × 200 mm², acquisition matrix = 64 × 64, flip angle = 90°, 33 axial slices, slice thickness = 3.5 mm, and interslice gap = 0.7 mm. A total of 200 functional volumes were obtained for each participant. High-resolution structural images were additionally acquired using a T1-weighted three-dimensional magnetization-prepared gradient echo sequence with TR = 2530 ms, TE = 1.85 ms, FOV = 256 × 256 mm², matrix = 256 × 256, flip angle = 9°, and slice thickness = 1 mm. The T1-weighted images were subsequently used to facilitate spatial normalization of the functional data.

### 2.4 Data preprocessing

Resting-state fMRI data were preprocessed using Statistical Parametric Mapping software (SPM12, revision 7487) and the Data Processing Assistant for Resting-State fMRI (DPARSFA, version 5.1). The first five volumes were removed, followed by slice timing correction and realignment. Nuisance covariates, including 24 head-motion parameters, the first five principal components of white matter and cerebrospinal fluid signals, and polynomial trends, were regressed out. To further reduce motion-related artifacts, scrubbing was performed within nuisance regression. Volumes with Power framewise displacement (FD) > 0.5 mm, together with one preceding and two subsequent volumes, were included as separate regressors in the nuisance regression model. The functional images were normalized to Montreal Neurological Institute (MNI) space based on the individual T1-weighted images, resampled to 2 × 2 × 2 mm³ voxels, spatially smoothed with a 4-mm full width at half maximum Gaussian kernel, and band-pass filtered at 0.01–0.10 Hz. After preprocessing, participants were excluded if head translation exceeded 2 mm, head rotation exceeded 2°, or mean FD exceeded 3 standard deviations above the sample mean. Based on these criteria, ten patients were excluded, leaving 87 patients for subsequent analyses: 42 in the RD group and 45 in the NRD group.

### 2.5 Functional connectivity analysis

The hippocampus was parcellated using the Scale III (S3) parcellation of the 3.0 T Melbourne Subcortex Atlas developed by Tian et al. (30). Each hippocampus was divided into the medial head, lateral head, body, and tail, yielding eight bilateral hippocampal subregions, which were separately used as seeds for rsFC analysis (**Figure 1**). In the 2 × 2 × 2 mm³ MNI space, the medial head, lateral head, body, and tail subdivisions contained 130, 216, 198, and 106 voxels, respectively, in both the left and right hippocampi. For each participant, the mean blood oxygen level-dependent (BOLD) time series was extracted from each hippocampal seed and correlated with the time series of every voxel within the whole-brain mask using Pearson correlation. The resulting correlation coefficients were converted to z values using Fisher’s r-to-z transformation and entered into subsequent group-level statistical analyses.

**Figure 1:**
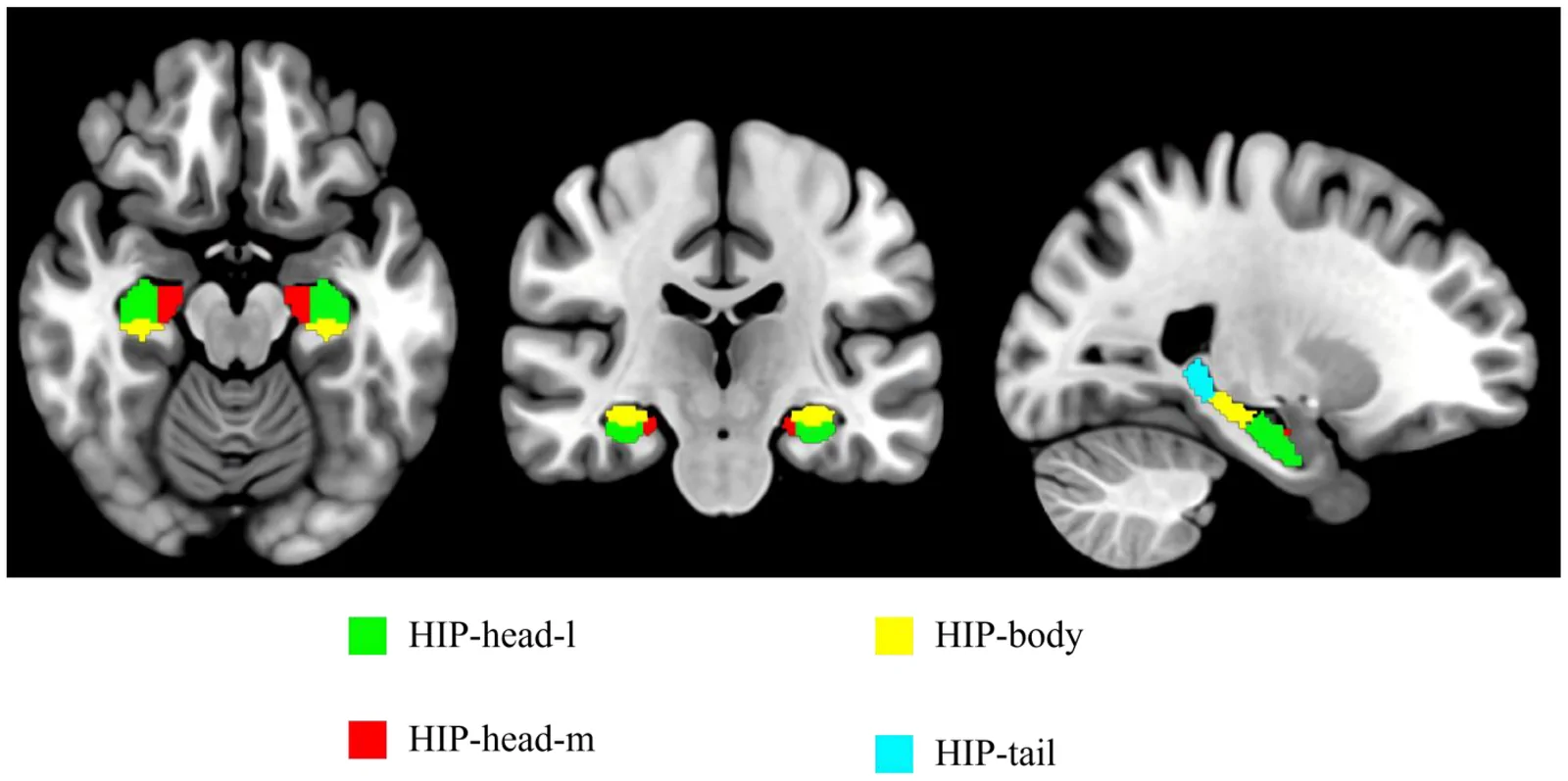
Hippocampal subregions defined using the Scale III (S3) parcellation of the 3-T Melbourne Subcortex Atlas. The bilateral medial head, lateral head, body, and tail were used as seed regions for whole-brain resting-state functional connectivity analyses.

### 2.6 Statistical analysis

Demographic and clinical data were analyzed using IBM SPSS Statistics, version 21.0. Continuous variables were expressed as the mean ± standard deviation, whereas categorical variables were presented as frequencies. Differences in continuous variables between the RD and NRD groups were assessed using independent two-sample *t*-tests or Mann–Whitney U tests, and categorical variables were compared using χ^2^ tests or Fisher’s exact tests, as appropriate. For the imaging analyses, each seed-based whole-brain rsFC map was tested separately using a two-sample t-test in SPM12 to determine whether connectivity involving an individual hippocampal seed differed between the RD and NRD groups. Age, sex, education level, and mean FD were included as covariates. The voxel-wise threshold was set at *p* < 0.001, with cluster-level false discovery rate (FDR) correction at *p* < 0.05 applied separately within each seed-based whole-brain rsFC map. To additionally control for multiple comparisons across the eight hippocampal seeds, a Bonferroni-adjusted threshold of *p_Bonf* < 0.00625 (0.05/8) was applied. Mean functional connectivity values were extracted from clusters showing significant between-group differences for subsequent analyses.

Within the MDD group, partial correlation analyses were performed to examine associations between baseline functional connectivity and the percentage reduction in HAMD-17 scores, controlling for age, sex, and education level. *p* values were corrected for multiple comparisons across the rsFC measures using the Benjamini–Hochberg FDR procedure. Exploratory binary logistic regression analyses were then performed with 12-week remission status as the outcome to examine the relationship between baseline rsFC and treatment outcome. Separate models were constructed for each rsFC measure, followed by a multivariable model combining all the connectivity measures that showed significant between-group differences. Exploratory receiver operating characteristic (ROC) curve analyses were used to assess the discriminative performance of the individual connectivity measures and the combined model. The area under the curve (AUC) with its 95% confidence interval (CI) was calculated, with CIs estimated using 2,000 bootstrap resamples. Sensitivity and specificity were calculated at a fixed threshold of 0.5 for the predicted probability of nonremission, with nonremission treated as the positive class. All statistical tests were two-sided, with *p* < 0.05 considered statistically significant.

## 3 Results

### 3.1 Demographic and clinical characteristics

As summarized in **Table 1**, the RD and NRD groups did not differ significantly in age, sex, education, illness duration, episode type, number of episodes, or baseline HAMD-17 scores. Following 12 weeks of treatment, the RD group had significantly lower HAMD-17 scores and a greater percentage reduction in HAMD-17 scores than the NRD group (both *p* < 0.001). Mean FD was significantly higher in the NRD group than in the RD group (0.159 ± 0.066 vs. 0.128 ± 0.059 mm, *p* = 0.025) and was included as a covariate in the between-group rsFC analyses. Only one patient in the RD group reported using zopiclone for insomnia, whereas no patient in the NRD group reported using medication for insomnia (*p* = 0.483).

**Table 1:** Demographic and clinical characteristics of patients in the RD and NRD groups.

Variable	RD (n = 42)	NRD (n = 45)	*t/χ^2^*	*p* value
Age, years	27.14 ± 5.84	27.00 ± 6.86	0.10	0.917
Sex, male/female	16/26	12/33	1.30	0.254
Education level, n			1.49	0.475
High school or college	8	7	-	-
Undergraduate	26	33	-	-
Postgraduate or above	8	5	-	-
Illness duration, montds	27.45 ± 33.72	45.67 ± 67.78	−1.60	0.114
Episode type, n				
first/recurrent	25/17	31/14	0.83	0.381
Number of episodes	1.67 ± 1.12	1.80 ± 1.84	−0.41	0.687
Baseline HAMD-17 score	19.86 ± 3.71	21.53 ± 4.13	−1.99	0.050
Week-12 HAMD-17 score	4.38 ± 2.10	13.40 ± 4.47	−12.18	< 0.001
HAMD-17 reduction, %	77.09 ± 11.49	35.97 ± 24.19	10.23	< 0.001
Use of medication for insomnia, n (%)	1 (2.4)	0 (0.0)	-	0.483
Mean FD, mm	0.128 ± 0.059	0.159 ± 0.066	-2.285	0.025

RD, remission depression; NRD, nonremission depression; HAMD-17, 17-item Hamilton Depression Rating Scale; FD, framewise displacement.

### 3.2 Group differences in hippocampal subregion functional connectivity

After controlling for age, sex, education level, and mean FD, three hippocampal rsFC measures showed significant between-group differences at the seed-wise corrected threshold (voxel-wise *p <* 0.001, cluster-level *FDR-corrected p <* 0.05). Compared with the NRD group, the RD group showed significantly higher pretreatment rsFC between the left hippocampal tail and the right inferior parietal lobule, but significantly lower pretreatment rsFC between the right hippocampal body and the left middle occipital gyrus and between the right hippocampal tail and the right cuneus. After additional Bonferroni correction across the eight hippocampal seeds (*p_Bonf* < 0.00625), none of the identified functional connectivity differences remained statistically significant (**Table 2**; **Figure 2**).

**Table 2:** Hippocampal functional connectivity differences between the RD and NRD groups at the seed-wise corrected threshold.

Seed region	Region	Peak MNI coordinates (x, y, z)	Cluster size (voxels)	*t* value	Cluster-level *p_FDR*
R HIP-b	L MOG	−42, −86, 4	132	−4.48	0.031
L HIP-t	R IPL	48, −42, 54	156	4.46	0.012
R HIP-t	R CUN	18, −74, 24	159	−4.51	0.018

HIP-b, hippocampal body; HIP-t, hippocampal tail; MOG, middle occipital gyrus; IPL, inferior parietal lobule; CUN, cuneus; L, left; R, right; MNI, Montreal Neurological Institute; FDR, false discovery rate.

**Figure 2:**
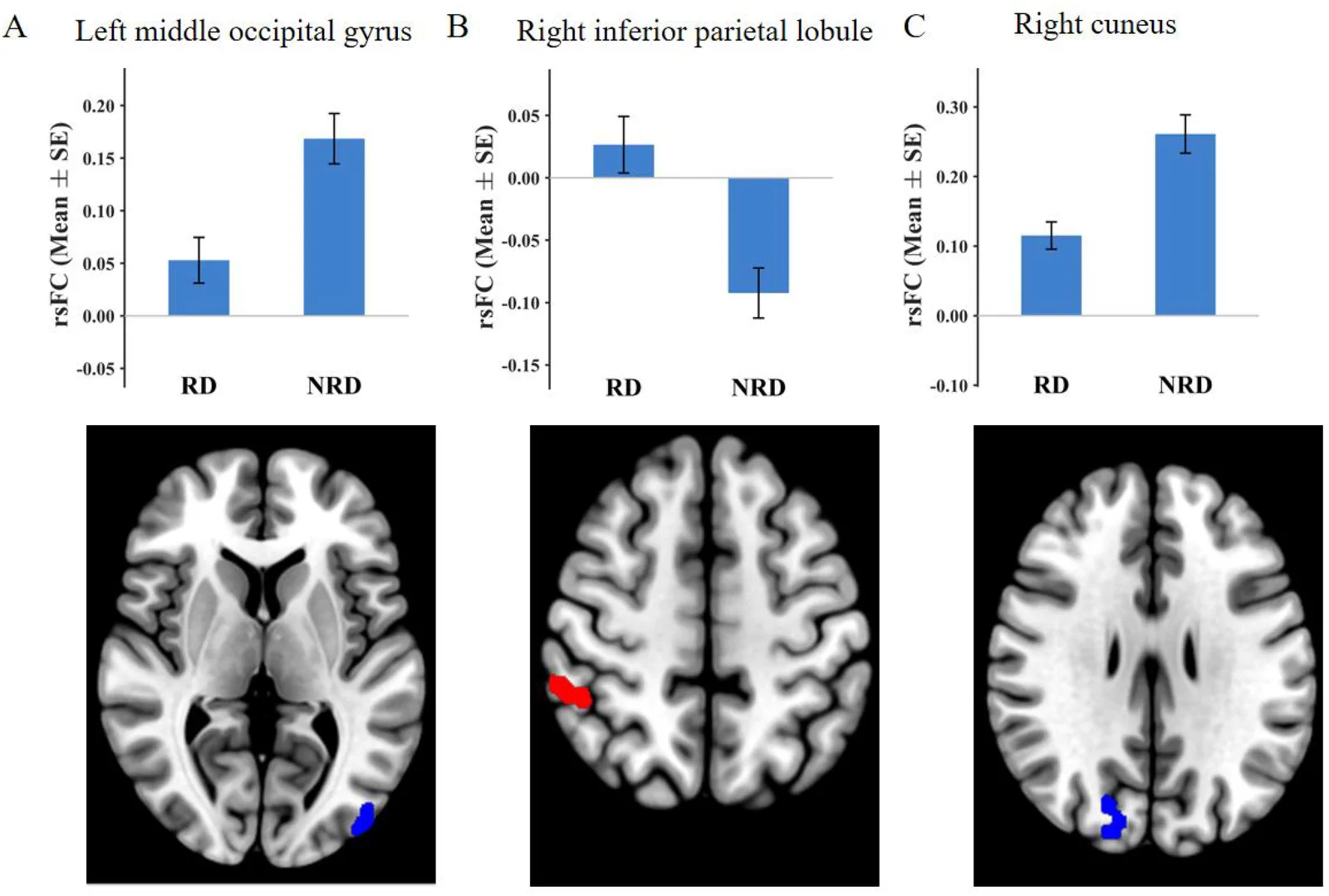
Group differences in hippocampal subregion-based rsFC. **(A)** right hippocampal body–left middle occipital gyrus; **(B)** left hippocampal tail–right inferior parietal lobule; **(C)** right hippocampal tail–right cuneus. Bar plots show mean rsFC values ± SE. rsFC, resting-state functional connectivity; RD, remission depression; NRD, nonremission depression; SE, standard error.

### 3.3 Associations of hippocampal subregion rsFC with symptom improvement

After adjustment for age, sex, and education level, partial correlation analyses were conducted to examine the associations between the three differential rsFC measures and the percentage reduction in HAMD-17 scores, with Benjamini–Hochberg FDR correction. A greater percentage reduction in HAMD-17 scores was negatively associated with rsFC between the right hippocampal body and the left middle occipital gyrus (*partial r =* −0.262*, p_FDR =* 0.016) and between the right hippocampal tail and the right cuneus (*partial r =* −0.303*, p_FDR =* 0.008), whereas it was positively associated with rsFC between the left hippocampal tail and the right inferior parietal lobule (*partial r =* 0.422*, p_FDR <* 0.001) (**Figure 3**).

**Figure 3:**
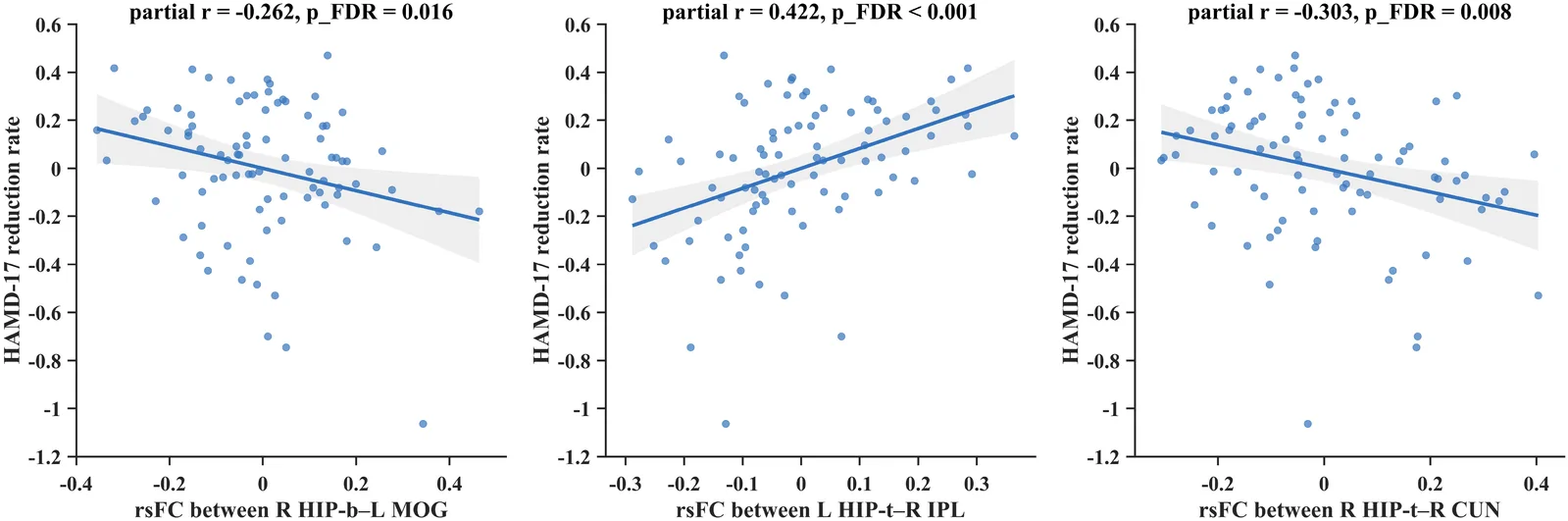
Results of partial correlation analyses. Scatter plots showing partial correlations between HAMD-17 reduction rate and rsFC, with negative correlations for R HIP-b–L MOG and R HIP-t–R CUN and a positive correlation for L HIP-t–R IPL. The plotted values represent residuals after regressing out age, sex, and education level from both rsFC values and HAMD-17 reduction rates. HAMD-17, 17-item Hamilton Depression Rating Scale; rsFC, resting-state functional connectivity; HIP-b, hippocampal body; HIP-t, hippocampal tail; MOG, middle occipital gyrus; IPL, inferior parietal lobule; CUN, cuneus; L, left; R, right; FDR, false discovery rate.

### 3.4 Discriminative performance of hippocampal subregion rsFC for remission

In the exploratory ROC analyses, the three individual rsFC measures showed discriminative ability between the RD and NRD groups, with AUC values ranging from 0.689 to 0.731 (all *p* < 0.001). An exploratory multivariable logistic regression model incorporating the three differential rsFC measures yielded an AUC of 0.841 (95% CI: 0.755–0.915) for distinguishing the RD group from the NRD group (**Table 3**; **Figure 4**).

**Table 3:** Exploratory ROC analysis of the individual rsFC models and the combined model.

rsFC	AUC (95% CI)	Sensitivity (%)	Specificity (%)	*p* value
R HIP-b–L MOG	0.689 (0.571-0.789)	66.7	57.1	<0.001
L HIP-t–R IPL	0.722 (0.612-0.821)	71.1	61.9	<0.001
R HIP-t–R CUN	0.731 (0.623-0.834)	62.2	71.4	<0.001
Combined rsFC model	0.841 (0.755-0.915)	77.8	69.0	<0.001

HIP-b, hippocampal body; HIP-t, hippocampal tail; MOG, middle occipital gyrus; IPL, inferior parietal lobule; CUN, cuneus; L, left; R, right; rsFC, resting-state functional connectivity; ROC, receiver operating characteristic; AUC, area under the curve; CI, confidence interval.

**Figure 4:**
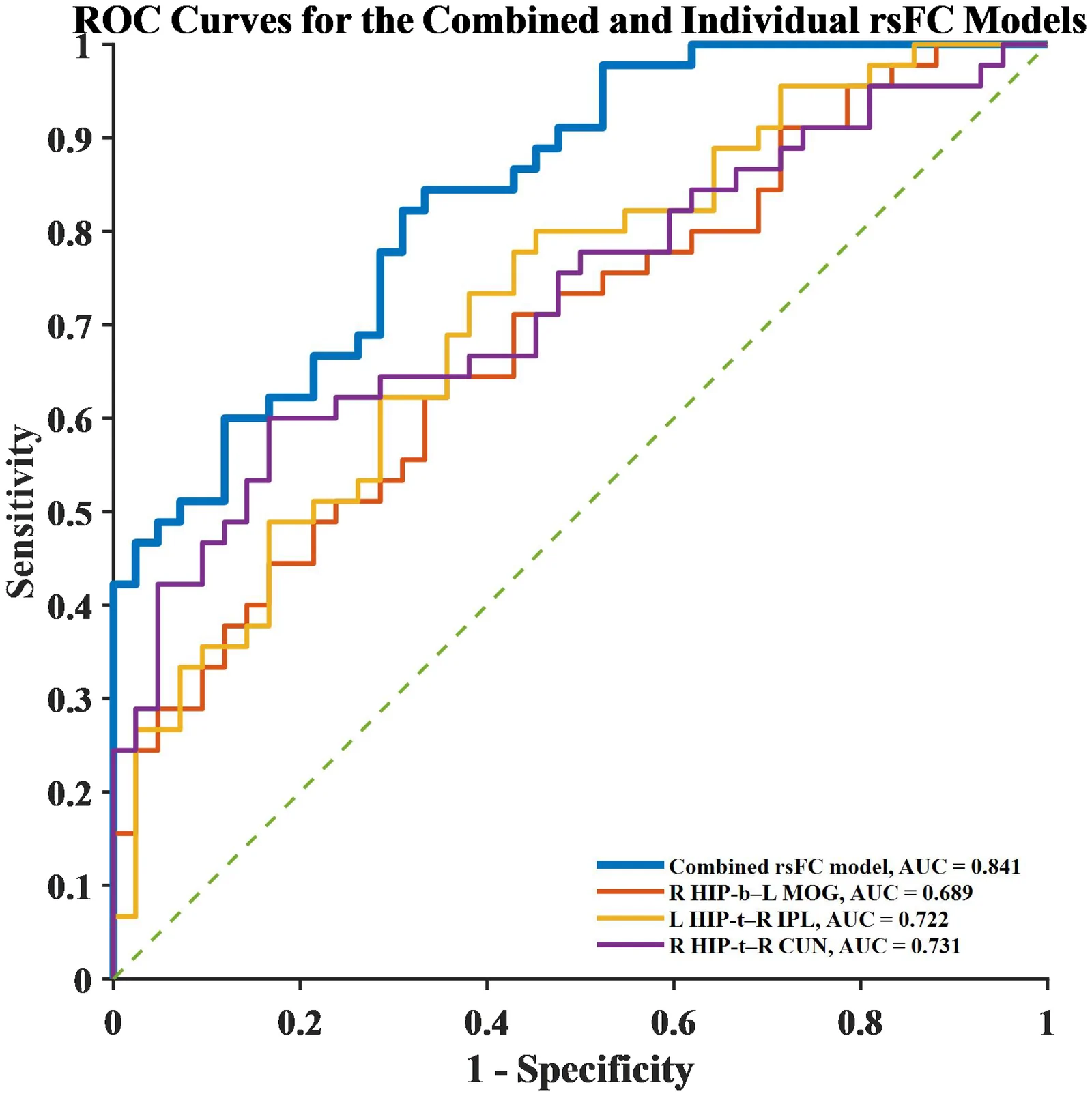
ROC analysis of remission discrimination. ROC curves show the discriminative performance of the three individual rsFC models and the combined model incorporating all three rsFC measures for 12-week remission status. ROC, receiver operating characteristic; rsFC, resting-state functional connectivity; AUC, area under the curve; HIP-b, hippocampal body; HIP-t, hippocampal tail; MOG, middle occipital gyrus; IPL, inferior parietal lobule; CUN, cuneus; L, left; R, right.

## 4 Discussion

Using resting-state functional MRI, this study investigated the relationship between pretreatment rsFC involving hippocampal subregions and 12-week escitalopram treatment outcomes in patients with MDD. After adjustment for age, sex, education, and mean FD, the RD group exhibited higher baseline rsFC between the left hippocampal tail and the right inferior parietal lobule, but lower baseline rsFC between the right hippocampal body and the left middle occipital gyrus and between the right hippocampal tail and the right cuneus, compared with the NRD group. These differences survived seed-wise FDR correction but not additional Bonferroni correction across the eight seeds. In an exploratory analysis, a combined logistic regression model based on these three differential rsFC measures yielded an AUC of 0.841 for distinguishing RD from NRD in the current sample. Overall, these findings suggest that pretreatment connectivity of specific hippocampal subregions with parietal and occipital regions is associated with subsequent remission status and symptom improvement during escitalopram treatment.

The present study found that pretreatment rsFC between the right hippocampal body and the left middle occipital gyrus was lower in the RD group than in the NRD group and was negatively associated with the percentage reduction in HAMD-17 scores, indicating that weaker baseline connectivity was associated with more favorable escitalopram treatment outcomes. Previous research showed that female patients with MDD exhibited reduced spontaneous activity in the left middle occipital gyrus but increased functional connectivity between this region and the left hippocampus, suggesting that hippocampal–middle occipital coupling forms part of the functional network abnormalities associated with MDD (32). In a study of antidepressant outcomes, baseline dynamic network features involving the right middle occipital gyrus and left superior occipital gyrus contributed to distinguishing escitalopram responders from nonresponders (33). Longitudinal studies further showed that a single dose of escitalopram reduced occipital activity, with greater early changes in remitters than in nonresponders (34), while ECT increased rsFC between the emotional hippocampal subregion and the left middle occipital gyrus (23). Taken together, previous studies have primarily demonstrated treatment-related changes in occipital activity and hippocampal–occipital connectivity, whereas the present findings indicate that individual differences in pretreatment hippocampal–occipital connectivity are also associated with subsequent treatment outcomes.

Compared with the NRD group, the RD group exhibited greater pretreatment rsFC between the left hippocampal tail and the right inferior parietal lobule, and stronger connectivity was associated with a greater HAMD-17 reduction rate. These findings suggest that higher baseline hippocampal tail-inferior parietal lobule connectivity is associated with more favorable escitalopram treatment outcomes. The inferior parietal lobule is a higher-order association cortical region involved in attention, memory, and complex cognitive processing (35). Previous work has shown functional connectivity between the hippocampal tail and the parietal memory network, which is implicated in goal-directed cognition and stimulus recognition (36). Neuromodulation and intracranial electrophysiological studies also support functional interactions between the hippocampus and parietal cortex: stimulation of lateral parietal regions with strong functional connectivity to the hippocampus enhances cortico-hippocampal network connectivity and is accompanied by improvements in associative memory (37), while intracranial recordings demonstrate frequency-dependent and directional interactions between the hippocampus and lateral parietal cortex (38). In studies of antidepressant outcomes, remitters have shown greater pretreatment inferior parietal lobule activation than nonremitters during response inhibition (39). Xiao et al. (18) further reported that patients showing early improvement following escitalopram treatment exhibited higher pretreatment rsFC between the left hippocampus and the precuneus, a region located in the parietal lobe, with stronger connectivity associated with greater symptom reduction. Similarly, Zhang et al. (25) found that patients who achieved remission following repeated ketamine infusions showed less negative pretreatment functional connectivity of the left rostral hippocampus with parietal regions, including the right angular gyrus, left inferior parietal cortex, and right superior parietal cortex, than nonremitters. Although previous studies differ from the present study in experimental paradigms and neuroimaging measures, the available evidence suggests that parietal functional features may be implicated in MDD pathophysiology and clinical improvement following antidepressant treatment. However, the specific role of this functional coupling in antidepressant response remains unclear, and its clinical relevance requires further validation in longitudinal studies and independent cohorts.

In addition, RD exhibited lower pretreatment rsFC between the right hippocampal tail and the right cuneus than NRD, and stronger connectivity was associated with a lower HAMD-17 percentage reduction. The cuneus, located within the medial occipital cortex, is an integral component of the visual processing system and contributes to both basic and higher-order visual processing (40). Accumulating evidence has pointed to functional abnormalities within the visual system. For example, reduced regional homogeneity in the right cuneus has been reported in patients with first-episode, drug-naïve MDD (41). At the network level, a large-scale connectomic study identified hyperconnectivity between the visual network and the default mode, frontoparietal, ventral attention, and limbic networks, with several cross-network connections associated with depressive symptom severity (42). Consistent with the direction of the present findings, first-episode, drug-naïve adolescents who did not respond to SSRI treatment showed higher pretreatment betweenness centrality in the left cuneus, which was negatively associated with HAMD-17 score reduction (43). Together, these findings suggest that greater pretreatment functional integration of the cuneus, reflected by either higher network centrality or stronger hippocampal tail connectivity, may be associated with less subsequent symptom improvement.

Notably, two of the three treatment outcome-related rsFC differences identified in this study involved hippocampal tail seeds. Previous studies have also implicated the posterior hippocampus or hippocampal tail in antidepressant treatment outcomes in MDD. MacQueen et al. (44) found that remitters had larger pretreatment bilateral hippocampal body and tail volumes than nonremitters, whereas hippocampal head volumes did not differ between the groups. Maller et al. (45) found that larger baseline hippocampal tail volume was associated with clinical remission following antidepressant treatment, independently of total hippocampal volume. In an independent subsequent study, Nogovitsyn et al. (46) further showed that larger baseline hippocampal tail volume was associated with remission at both weeks 8 and 16, and that patients with early sustained remission had larger hippocampal tail volumes than those with persistent nonremission. Functional studies have further implicated posterior hippocampal connectivity in subsequent symptom improvement. Higher pretreatment global connectivity in posterior hippocampal nodes contributed to the prediction of greater symptom improvement after 8 weeks, regardless of whether patients received sertraline or placebo (47). In addition, individualized functional parcellation revealed reduced connectivity between the left hippocampal tail and the right primary visual cortex in patients with MDD; this connectivity increased toward levels observed in healthy controls following ECT, and higher post-ECT connectivity was associated with lower depression and anxiety severity (48). Together, these structural and functional findings suggest that the posterior hippocampus, particularly the hippocampal tail, may be relevant to subsequent clinical improvement. The present findings further support examining hippocampal subregions and their connections with different cortical regions separately when investigating treatment outcomes in MDD.

Several limitations should be acknowledged. First, this was a single-center study with a relatively limited sample size. Although none of these differences survived additional Bonferroni correction across the eight hippocampal seeds, their concordant associations with both remission status and HAMD-17 reduction provide preliminary evidence that these hippocampal-cortical connectivity patterns may be relevant to treatment outcomes. Second, because the three rsFC measures were selected and evaluated in the same sample and no internal validation was performed, the reported AUC, sensitivity, and specificity may be optimistic. Their performance therefore requires further evaluation using more rigorous validation procedures. Third, the eligibility and treatment-related exclusion criteria may have limited the representativeness of the study sample. Patients with a history of intolerance or nonresponse to escitalopram were not eligible, and those who violated the monotherapy protocol or experienced serious adverse drug reactions during treatment were withdrawn. In addition, although treatment was managed by experienced psychiatrists according to a predefined dosing framework, subsequent dose adjustments were individualized within the therapeutic range, and medication adherence was assessed primarily through patient self-report. Therefore, suboptimal adherence and residual variability in treatment exposure cannot be completely excluded. Fourth, the study investigated escitalopram monotherapy without an active treatment comparison group. It therefore remains unclear whether the identified hippocampal connectivity features are specific to escitalopram or reflect more general prognostic markers of antidepressant treatment outcomes. Comparative studies involving different treatment modalities are needed to address this question. Fifth, the spatial resolution of the functional images and 4-mm smoothing may have introduced partial volume effects and signal mixing between adjacent hippocampal subregions, warranting caution in interpreting the subregional localization of the findings. Finally, the analyses were based on baseline rsFC. Longitudinal neuroimaging studies incorporating additional clinical and imaging measures may provide a more comprehensive characterization of the neural mechanisms underlying individual differences in treatment outcomes.

In conclusion, the present study suggests that pretreatment hippocampal subregion functional connectivity may be associated with 12-week escitalopram treatment outcomes in patients with MDD. Compared with the NRD group, the RD group exhibited higher rsFC between the left hippocampal tail and the right inferior parietal lobule, but lower rsFC between the right hippocampal body and the left middle occipital gyrus and between the right hippocampal tail and the right cuneus. Left hippocampal tail–right inferior parietal lobule rsFC was positively associated with the percentage reduction in HAMD-17 scores, whereas the other two rsFC measures were negatively associated with symptom reduction. An exploratory model combining these three rsFC measures showed discrimination between remission and nonremission within the current sample. Collectively, these findings provide preliminary evidence that pretreatment connectivity involving hippocampal subregions may help characterize individual differences in clinical outcomes during escitalopram treatment.

## Data Availability

The datasets presented in this article are not readily available due to participant privacy and ethical restrictions. Access may be considered upon reasonable request to the corresponding authors, subject to ethics approval and institutional requirements.
